# Are mesenchymal stem cells in rheumatoid arthritis the good or bad guys?

**DOI:** 10.1186/s13075-015-0634-1

**Published:** 2015-05-01

**Authors:** Cosimo De Bari

**Affiliations:** Regenerative Medicine Group, Musculoskeletal Research Programme, Institute of Medical Sciences, University of Aberdeen, Foresterhill, Aberdeen, AB25 2ZD UK

## Abstract

The advancements in our understanding of the inflammatory and immune mechanisms in rheumatoid arthritis (RA) have fuelled the development of targeted therapies that block cytokine networks and pathogenic immune cells, leading to a considerable improvement in the management of RA patients. Nonetheless, no therapy is curative and clinical remission does not necessarily correspond to non-progression of joint damage. Hence, the biomedical community has redirected scientific efforts and resources towards the investigation of other biological aspects of the disease, including the mechanisms driving tissue remodelling and repair. In this regard, stem cell research has attracted extraordinary attention, with the ultimate goal to develop interventions for the biological repair of damaged tissues in joint disorders, including RA. The recent evidence that mesenchymal stem cells (MSCs) with the ability to differentiate into cartilage are present in joint tissues raises an opportunity for therapeutic interventions via targeting intrinsic repair mechanisms. Under physiological conditions, MSCs in the joint are believed to contribute to the maintenance and repair of joint tissues. In RA, however, the repair function of MSCs appears to be repressed by the inflammatory milieu. In addition to being passive targets, MSCs could interact with the immune system and play an active role in the perpetuation of arthritis and progression of joint damage. Like MSCs, fibroblast-like synoviocytes (FLSs) are part of the stroma of the synovial membrane. During RA, FLSs undergo proliferation and contribute to the formation of the deleterious pannus, which mediates damage to articular cartilage and bone. Both FLSs and MSCs are contained within the mononuclear cell fraction *in vitro*, from which they can be culture expanded as plastic-adherent fibroblast-like cells. An important question to address relates to the relationship between MSCs and FLSs. MSCs and FLSs could be the same cell type with functional specialisation or represent different functional stages of the same stromal lineage. This review will discuss the roles of MSCs in RA and will address current knowledge of the relative identity between MSCs and FLSs. It will also examine the immunomodulatory properties of the MSCs and the potential to harness such properties for the treatment of RA.

## Introduction

Extensive investigations of the pathogenetic mechanisms of inflammation and autoimmunity and the resulting increased understanding of cytokine networks and cellular players in rheumatoid arthritis (RA) have led to the development of agents that block tumour necrosis factor (TNF)α, interleukin (IL)-1 and IL-6 signalling, or target pathogenic cells such as B cells and osteoclasts [1,2]. Despite significant therapeutic advances, however, two major problems remain unresolved: (i) up to 30% of RA patients fail to respond to treatments [1], and (ii) radiographic progression of joint damage can occur even when clinical remission of the inflammatory component of the disease is achieved [3,4]. Mechanisms of joint destruction appear to be at least in part uncoupled from inflammation [5]; hence, suppression of inflammation may not be sufficient to stop RA disease progression.

A hallmark of RA joint pathology is chronic inflammation of the synovium (synovitis), which causes cartilage and bone erosion via interplay between infiltrating inflammatory/immune cells and the resident fibroblast-like synoviocytes (FLSs). Once established, the erosions do not heal, posing considerable risks for joint damage progression towards secondary osteoarthritis and joint failure. The synovium is also home to mesenchymal stromal/stem cells (MSCs) [6-9]. These cells, among other functions, are thought to maintain tissues in adult life and participate in repair processes. While both FLSs and MSCs are part of the stroma of the synovium, their relationship remains unclear. FLSs and MSCs could be the same cell type with functional specialisation and diversification according to their positional information and environmental cues, or they could represent different functional stages of the same lineage. This review will cover recent insights into the roles of MSCs in RA while addressing current knowledge of the relative identity between MSCs and FLSs, and will discuss the potential to harness the immunomodulatory properties of MSCs for the treatment of RA.

## The stroma of synovium: not a one-fibroblast-fits-all

A key tissue in RA is the synovium, a membrane that lines the cavity of synovial joints. The synovium lubricates the joint surfaces and provides nutrients to the articular cartilage. It consists of a lining layer of macrophage-like (type A) synoviocytes and FLSs (type B synoviocytes), and a sublining of loose connective tissue containing fibroblasts interspersed between endothelium (with juxtaposed pericytes) of small blood vessels. The fibroblasts appear to be functionally distinct depending on their location. FLSs in the synovial lining share with the common fibroblasts many characteristics, including expression of type IV and V collagens, vimentin and CD90 (Thy-1). However, they have distinctive features from other fibroblasts, including the fibroblasts resident in the synovial sublining whose main function is thought to be production and remodelling of extracellular matrix [10]. The FLSs in the synovial lining express uridine diphosphoglucose dehydrogenase to synthesise hyaluronan, an important constituent of synovial fluid, and secrete lubricin, another critical protein for joint lubrication [10]. Furthermore, FLSs express cadherin-11, an adhesion molecule that plays a key role in homotypic aggregation of FLSs *in vitro* and *in vivo* [11,12]. FLSs, but not dermal fibroblasts, have the ability to reproduce a lining-like structure in a three-dimensional culture *in vitro* with similarity to the synovial lining *in vivo* [13]. Cadherin-11-deficient mice develop normally but lack a defined synovial lining. In addition, cadherin-11 null FLSs fail to develop a lining-like structure *in vitro*, indicating that lining layer condensation is an inherent feature of FLSs that requires cadherin-11 [12]. Thus, FLSs in the lining are a specialised subgroup of fibroblasts, which can be recognised for their position and expression of cadherin-11, and appear to be functionally distinct from the fibroblasts in the sublining stroma.

Recent lineage tracing studies in mice have unveiled that, like articular cartilage, the synovium derives from the embryonic joint interzone [14,15], a stripe of mesenchymal tissue in the developing limbs located at the site of the prospective joint. The joint interzone consists of two perichondrium-like chondrogenic layers and one intermediate narrow band of mesenchymal cells. The central layer of the interzone undergoes a cavitation process with the appearance of small clefts which extend and coalesce to form the synovial cavity [16]. Cells of the interzone then give rise to the synovium, as well as other joint structures, including articular cartilage, menisci and ligaments [14,15]. However, whether every single cell in the synovium originates from the joint interzone is not known. Macrophages and endothelial cells are unlikely to descend from the joint interzone and instead are most likely to derive from the bone marrow [17]. With regards fibroblasts, we could postulate a dual origin, with FLSs of the lining being progeny of the joint interzone and the fibroblasts of the sublining possibly deriving from the bone marrow or, more generally, blood-borne fibroblasts. In this regard, third passage primary FLS cultures established from normal synovial joints of mice carrying green-fluorescent protein (GFP)-positive bone marrow comprised approximately 1% of GFP-positive (bone marrow-derived) fibroblast-like cells [18]. Distinct origins of the synovial fibroblast populations may be the basis of functional differences and would strengthen the notion that the FLSs of the lining and the fibroblasts of the sublining are distinct cell types. The modern technologies of lineage tracing will shed light on the origins of the fibroblasts in the synovium.

## Mesenchymal stem cells in synovium: a new stromal cell player or an old fibroblast?

MSCs were originally isolated from bone marrow [19]. In 2001, we reported the isolation and characterisation of multipotent MSCs from the adult human synovium [6]. MSCs *in vitro* are fibroblast-like cells capable of plastic adherence, form colonies derived from single cells (colony forming unit fibroblasts) and can differentiate into mature cells of mesenchymal lineages such as osteoblasts and chondrocytes [19-22]. The discovery that the adult human synovium contains cells that after isolation and culture-expansion display a MSC phenotype and perform MSC functions inspired the intriguing speculation that, postnatally, the synovium may function as a reservoir of stem cells for the regeneration or repair of joint tissues such as the articular cartilage, which have limited intrinsic repair potential [16]. Of note, in a comparative study of MSCs from multiple tissue sources, including bone marrow, the synovial MSCs were superior in cartilage formation [23], suggesting that they may be the 'natural' chondroprogenitors for articular cartilage repair.

Following enzymatic release from the synovium, MSCs and FLSs are both contained within the plastic-adherent mononuclear cell fraction *in vitro*, from which they can be culture-expanded as fibroblast-like cells. Cultures of FLSs and MSCs are therefore indistinguishable, and at present no markers permit selective identification of either cell type from culture-expanded synovial stromal cell populations. It is not known, therefore, whether FLS and MSC properties reside in the same individual cell or in distinct cell types.

To shed light on the relationship between these two cell types, we carried out studies at the single cell level. Culture-expanded synovial clonal cell populations from normal or osteoarthritic donors displayed a phenotype compatible with conventional bone marrow MSCs [24]. However, markers alone would not be sufficient to rule out the presence of FLSs or fibroblasts in general, as culture conditions are known to affect cell phenotype. All the 21 synovial cell clones obtained and tested from six donors were capable of chondrogenic and osteogenic differentiation, while only 30% of the clones were adipogenic [24]. Since all clones displayed mesenchymal differentiation potency, one could argue that the MSC property would be inherent to each plastic-adherent cell, at least after *in vitro* culture expansion. However, the extensive culture expansion required to perform all the necessary tests to investigate the mesenchymal potency may have selected for MSC clones, while FLSs or other fibroblasts were left behind. In addition, primary fibroblasts derived from various human tissues, including skin, were reported to contain cells that were able to differentiate into osteoblasts, chondrocytes and adipocytes [25].

Primary cultures of plastic-adherent cells from RA synovium (commonly regarded as FLSs) have been shown to contain cells with the functional ability, typical of RA FLSs, to erode cartilage through matrix metalloproteinases [17,26], as well as cells with the typical mesenchymal multipotency of MSCs [27,28]. The relationship between MSCs and FLSs in the synovial cell pool *in vitro* is yet to be clarified, and studies using single cell-derived clonal populations will be needed to determine whether FLS invasiveness and MSC differentiation potency are inherent in individual cells from the RA synovium.

Recently, we reported the *in vivo* identification and location of MSCs in mouse synovium [29]. We developed a double-nucleoside analogue labelling method to identify functional MSCs *in situ* in the knee joints of mice [29] to overcome the hurdle of a lack of MSC-specific markers. Our labelling approach relied on the slow-cycling nature of MSCs combined with their propensity to undergo proliferation following joint surface injury. Nucleoside-labelled cells were non-haematopoietic, non-endothelial stromal cells which expressed known MSC markers and formed ectopic cartilage following joint surface injury and patellar dislocation [29], thereby demonstrating that these cells have the ability to function as MSCs in their native environment.

**Figure 1:**
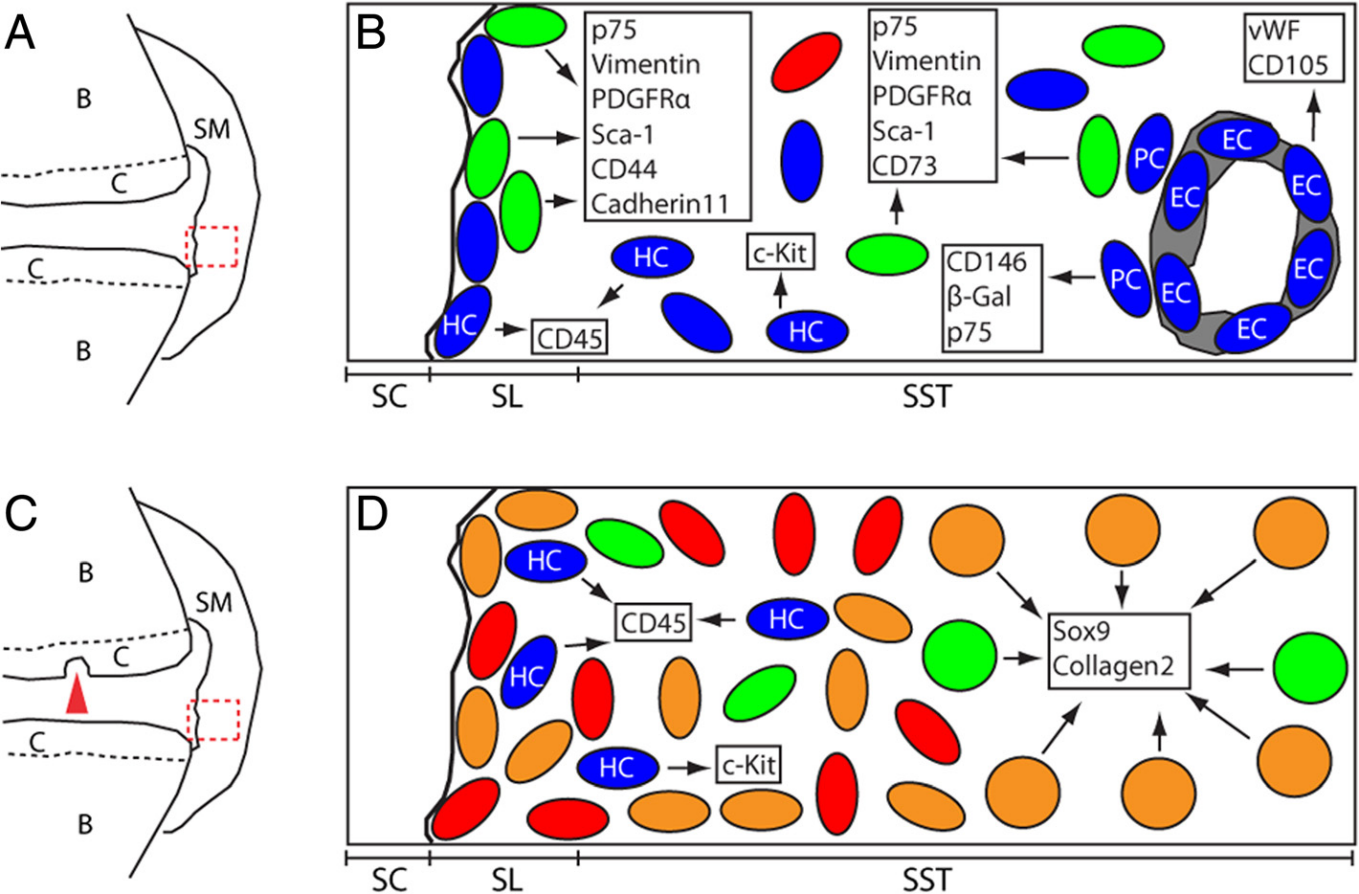
Schematic representations of mesenchymal stem cells (MSCs) and their niches in synovium identified in mice using a double-nucleoside analogue cell-labelling scheme [29]. **(A)** Schematic drawing of an uninjured control synovial joint. **(B)** Details of the dashed box in **(A)**, showing cell populations in the synovium of uninjured joints. Iododeoxyuridine (IdU)-retaining cells (green) were located in both the synovial lining (SL) and the subsynovial tissue (SST). Subsets of IdU-positive cells displayed an MSC phenotype. IdU-negative cells (blue) included haematopoietic lineage cells (HC), endothelial cells (EC), pericytes (PC), and other cell types of unknown phenotype. **(C)** Schematic drawing of a synovial joint 12 days after articular cartilage injury in mice (arrowhead). **(D)** Details of the dashed box in **(C)**, showing cell populations in the synovium. Proliferating cells were detected in both the synovial lining and the subsynovial tissue and were either double positive for IdU and chlorodeoxyuridine (CIdU; orange) or single positive for CIdU (red). Subsets of cells positive for IdU and CIdU and cells positive only for IdU (green) expressed chondrocyte lineage markers. The boxed areas in **(B)** and **(D)** show cell phenotypes. B, bone; C, cartilage; SC, synovial cavity; SM, synovial membrane. Reproduced from Kurth *et al*., Arthritis Rheum 2011 [29].

In synovium, MSCs are located mainly in two niches (Figure 1): the lining niche and the sublining perivascular niche, the latter distinct from pericytes [29]. In these two niches, MSCs could have distinct functions and still be geographically interchangeable, but a temporo-spatial hierarchy between the two MSC niches remains to be investigated. Furthermore, MSCs in synovium are heterogeneous in their phenotype, and this could possibly reflect a coexistence of functionally distinct cell subsets [29]. At present, the developmental origins of MSCs in the adult synovium are not known. They could derive from the embryonic joint interzone but a contribution from blood-borne circulating MSCs into the synovial pool would not be surprising given that MSCs can be found in the circulation [30] and are likely to traffic across, home to and engraft in tissues and organs of the entire body. Origins may differ for MSCs found at distinct niche sites. The ontogeny of MSCs in synovium and their maintenance throughout life via possible contribution from other tissues such as bone marrow is an exciting area of investigation.

Meanwhile, the relationship between the MSCs and the FLSs in the lining layer remains unclear. In our study [29], label-retaining (slow-cycling) cells were positive for the MSC markers PDGFRα, p75/LNGFR, and CD44. However, CD44 is also known to be expressed by FLSs [31], and label-retaining cells in the lining layer co-stained for cadherin-11 [29], a known marker of FLSs [12]. MSCs in the lining could be 'professional' stem cells, interspersed in between the FLSs and the macrophages. Alternatively, the FLSs could be a stage of differentiation of the MSC lineage, attaining FLS-specific properties but perhaps remaining able to function as 'non-professional' MSCs under challenging circumstances, including joint injury or inflammation *in vivo*, or after isolation and culture expansion *in vitro*. The existence of cell plasticity and dedifferentiation has long been controversial, but the induced pluripotent cell technology [32] has provided 'extreme' proof-of-concept under specific experimental conditions. If such plasticity were to exist *in vivo*, it could allow cells to swing between the perhaps imprinted embryonic memories of MSCs and the tissue-specific, functionally specialised cells like the FLSs.

## Mesenchymal stem cells: good or bad in rheumatoid arthritis?

Our current knowledge of the roles of MSCs in RA is limited. MSCs appear to be passive targets of the inflammatory process but they could also play an active pathogenic role. While under homeostatic conditions the synovium contributes to joint maintenance, in RA this tissue exerts a deleterious, damaging action on the joint, and the FLSs are known to be major pathogenic cell players. During RA, the synovium forms a 'pannus' that invades and erodes cartilage and bone. The pannus is a pathological outgrowth of synovial tissue sustained mainly by proliferation of FLSs, with infiltration of blood-borne inflammatory/immune cells. There is also evidence suggesting an influx of mesenchymal cells from bone marrow into synovium. In this regard, primary FLS cultures established from RA-like arthritic joints of mice carrying GFP-positive bone marrow contained over 30% of GFP-positive (bone marrow-derived) cells, significantly higher than the approximately 1% observed in FLS cultures obtained from normal joints [18]. The molecular mechanisms underpinning such inflow of mesenchymal cells from bone marrow into synovium during inflammatory synovitis are not fully known but chemokines would likely play a role [33]. Recent work has demonstrated that placental growth factor, whose levels are increased in RA joints, could recruit bone marrow MSCs to the synovium, where the interactions with the resident FLSs would contribute to angiogenesis and chronic synovitis by enhancing further the secretion of placental growth factor [34].

The erosive changes that occur in association with the inflammatory synovitis in RA indicate prevalence of cartilage/bone loss over repair. FLSs are well known to produce inflammatory cytokines and to develop an invasive phenotype with release of proteases that cause cartilage and bone destruction [35]. At the same time, remodelling/reparative responses appear to be suppressed probably by the persistent inflammation. The prevalence of MSCs, as characterised by *in vitro* multilineage potential, was significantly lower in the synovial fluid of RA patients than osteoarthritis patients [36]. In addition, there was a negative relationship between synovial MSC chondrogenic and clonogenic capacities and the magnitude of synovitis in RA [28], suggesting a suppression of MSC repair function within the joint perhaps secondary to the high levels of inflammatory cytokines during RA. TNFα is indeed known to prevent the mesenchymal differentiation capabilities of MSCs *in vitro* [37,38]. Thus, in addition to the well-known catabolic effects of TNFα on articular cartilage and bone [1], TNFα signalling would decrease the reparative responses of endogenous joint MSCs, thereby limiting cartilage/bone regeneration during arthritis. Clinical studies in patients with RA indicate that targeting TNFα can result in inhibition of progression of structural joint damage [39].

In addition to being 'innocent bystanders' repressed in their stem cell function by the inflammatory milieu, MSCs in the joint could be active players contributing to the pathogenesis of arthritis. Inflammatory cytokines such as interferon (IFN)-γ are required *in vitro* to induce the immunosuppressive and anti-inflammatory functions in cultured MSCs [40], but whether MSCs in their native tissues *in vivo* exert such functions remains unknown. An intriguing possibility is that arthritic FLSs could be 'diseased' MSCs with a differentiation arrested at early stages, thereby becoming pathogenic cells actively contributing to RA chronicity and progression. A major downstream target of inflammatory cytokines is the transcription factor nuclear factor-κB, and its sustained activation in FLS/MSC cultures was sufficient to inhibit osteogenic and adipogenic differentiation and at the same time to enhance proliferation, motility, and matrix-degrading activity [12]. These findings would support the 'transformation hypothesis' that proposes that FLSs/MSCs become transformed by the chronic interplay with the inflammatory processes in the joint, resulting in a more aggressive cell type with the ability to invade the articular cartilage, as demonstrated in models of co-implantation of normal cartilage and RA FLSs *in vivo* in mice [26]. Notably, RA FLSs can circulate and spread arthritis to unaffected joints [41]. Thus, mesenchymal/stromal cell populations could contribute to initiation, maintenance and progression of arthritis, and would provide recruitment/retention and exit signals to other cell types, including immune cells [42].

## Culture-expanded mesenchymal stem cells as immunomodulatory therapy for rheumatoid arthritis

Alongside their stem cell properties, culture-expanded MSCs possess immunomodulatory properties. Studies predominantly using bone marrow-derived MSCs have demonstrated that MSC-mediated immunomodulation is dependent on IFN-γ [43], and is largely mediated by factors such as indoleamine 2,3-dioxygenase or nitric oxide synthase, inhibiting both T- and B-cell proliferation and function [44]. MSCs can also induce the differentiation of regulatory T cells and maintain their inhibitory function [45,46]. Furthermore, MSCs suppress innate immunity through inhibiting dendritic cell formation and function [47], decreasing the expression of human leukocyte antigen DR and CD80 and CD86 co-stimulatory molecules on antigen presenting cells [48], and decreasing the proliferation of both resting and IL-2-activated natural killer cells, their cytotoxic capabilities, and IFN-γ production [49]. The immunoregulatory properties of cultured synovial MSCs are less well known but the data available so far point to similar functions to their bone marrow counterparts [50-53].

**Figure 2:**
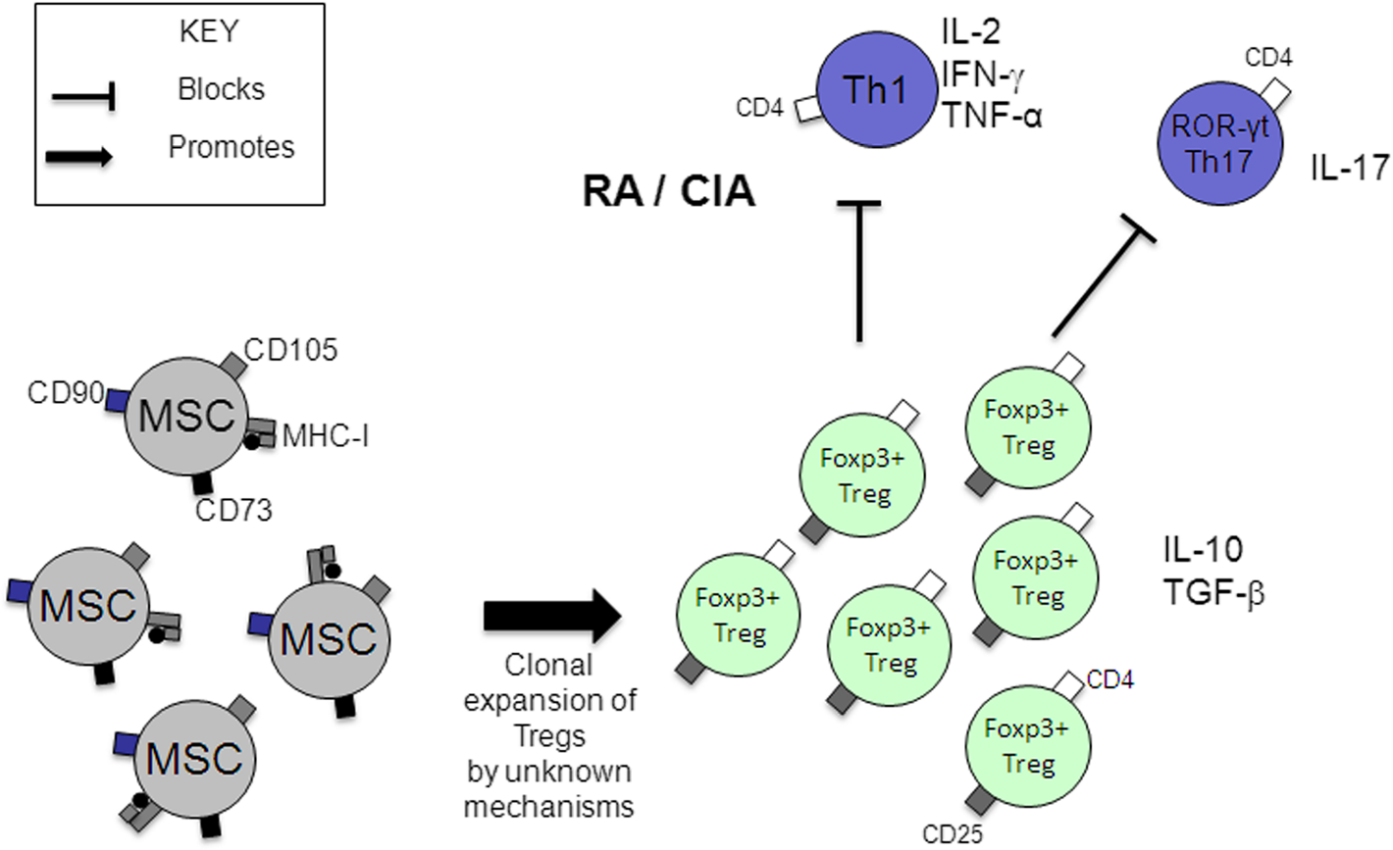
Possible effects of mesenchymal stem cells (MSCs) on regulatory T cell (Treg) and Th17 cell populations in rheumatoid arthritis (RA). CIA, collagen-induced arthritis; IFNγ, interferon-γ; IL-2, interleukin-2; MHC-I, class I major histocompatibility complex; RORγt, retinoic acid receptor-related orphan receptor γt; TGFβ, transforming growth factor β; TNFα, tumour necrosis factor α. Adapted from MacDonald *et al*., Arthritis Rheum 2011 [40].

The immunosuppressive and anti-inflammatory properties of cultured MSCs have led to these cells being tested for their therapeutic potential in preclinical models of RA-like inflammatory arthritis (reviewed in [40]). Several studies suggested that bone marrow- or adipose-derived MSCs have the ability to 'reset' the immune system by reducing the deleterious Th1/Th17 response and enhancing the protective regulatory T cell response (Figure 2), although other studies failed to demonstrate improvement with MSC treatment [40]. The inconsistent results in preclinical models may be due to several variables such as source of MSCs (murine syngeneic or allogeneic, or human), tissue of origin of MSCs, timing of treatment, number of cells injected, route of injection and treatment regimes, different culture conditions, as well as differences in mouse strains and animal housing conditions.

Meanwhile, clinical studies have also been carried out. Intravenous infusion of allogeneic bone marrow or umbilical cord MSCs into four patients with established RA resistant to disease-modifying antirheumatic drugs (DMARDs) and at least one anti-TNFα agent was safe and resulted in only partial and transient clinical improvement [54]. More recently, intravenous injection of umbilical cord MSCs in addition to DMARDs in 136 patients with active RA who had inadequate responses to traditional medication induced a significant clinical improvement when compared with the control group of 36 patients who received DMARDs plus medium without MSCs. The therapeutic effects were maintained for 3 to 6 months, and correlated with an increased percentage of regulatory T cells in peripheral blood [55]. Allogeneic MSCs could thus be effective in RA but a larger multi-centre clinical study will be needed to provide conclusive evidence. The use of MSCs in clinical studies is likely to be restricted to patients with severe RA refractory to standard therapies, but MSC treatment might be more effective if given at early stages of RA in order to 'reset' the immune system by inducing regulatory networks. The selection criteria of RA patients for such clinical studies will be crucial.

It is tempting to speculate that MSC treatment would control disease activity in RA patients not only through the immunosuppressive and anti-inflammatory functions but also through contribution to joint tissue repair, thereby preventing tissue damage, once established, from continuing to trigger inflammation. MSC therapeutic approaches to enhance joint tissue repair have been trialled in patients with joint surface defects and/or osteoarthritis with results that appear promising [56-61], supported by preclinical studies demonstrating cell engraftment and contribution to tissue formation leading to meniscal and cartilage repair [62-65]. Thus, the mechanisms through which MSCs can influence joint disease processes are diverse and include immunosuppressive and anti-inflammatory effects, trophic/paracrine effects and direct contribution to tissue repair. The elucidation of the mechanisms of action of MSC therapies will be critical to optimise cell product manufacturing for these positive effects, with the clinical goal of restoration of joint homeostasis likely to be crucial to halt disease progression.

## Immunomodulatory functions of native synovial fibroblast-like synoviocytes/ mesenchymal stem cells in joint homeostasis and rheumatoid arthritis

While immune cells have been extensively investigated in the pathogenesis of RA, little is known about the *in vivo* functions of FLSs/MSCs in the regulation of immune homeostasis in physiology and their contribution to immune deregulation in RA. It is possible that stromal cells in the synovium, particularly FLSs and MSCs, would be involved in the modulation of immune homeostasis within the healthy joint and that failure of such immunomodulation is the basis of RA development. While FLSs can inhibit T-cell proliferation [66] and the differentiation of monocytes into dendritic cells [67], similar to MSCs, RA FLSs have been shown to acquire class II major histocompatibility complex compared with healthy FLSs and work as antigen-presenting cells leading to T-cell activation and proliferation [68]. They can also induce the activation and accumulation of T cells following an interaction between CXCR4 on T cells and its ligand, stromal cell-derived factor-1 on RA FLSs [69]. RA FLSs can increase B-cell recruitment, survival and functions [70] and induce immunoglobulin class switching in B cells via B-cell activating factor and a proliferation-inducing ligand [71]. These findings suggest that within the RA inflammatory environment, MSCs/FLSs in synovium become unable to control inflammation and instead contribute to the perpetuation of the inflammation in concert with the aberrant immune system.

## Conclusions and future perspectives

Having discussed the multiple facets of MSCs in RA, from their potential role in the pathogenesis of RA, including their relationship with FLSs, to the possibility of using MSCs as immunomodulators for the treatment of RA, it becomes apparent that MSCs could be good or bad depending on the context.

Elucidation of the relationship between MSCs and FLSs will not only be an important scientific advancement, but will also lay the foundations for devising tailored therapeutic interventions for RA aiming at stopping the FLSs (bad MSCs) while stimulating the residual good MSC activity in the joint to achieve repair of damaged tissues such as cartilage and bone and restore joint homeostasis. The combination of modern research tools and technologies with pre-clinical mouse models of RA will be pivotal in addressing whether the FLSs are MSCs *per se* (and therefore a subset of the MSC pool) or are distinct specialised cells, likely down in the MSC lineage pathway. It will be interesting to determine whether FLSs/MSCs are descendants of the embryonic joint interzone; FLSs and MSCs could have distinct ancestors. These are some of the fundamental scientific questions that we and others are trying to address.

The interplay *in vivo* between FLSs/MSCs and immune cells in health and inflammatory arthritis also warrants further investigation. In normal conditions, FLSs/MSCs would control the degree of immune responses. Instead, during RA, due to the inflammatory environmental cues and the interplay with inflammatory/immune cells, the immunomodulatory functions of FLSs/MSCs are perturbed. FLSs/MSCs then proliferate, leading to the formation of the deleterious pannus with inflammatory and aggressive functions, thereby contributing to chronic disease maintenance and progression. Aberrant crosstalk between FLSs/MSCs and immune cells could be the basis of the vicious cycle underpinning RA chronicity and progression. An increased understanding of such crosstalk will be crucial to advance our targeted therapeutic armamentarium for RA patients to stop the vicious cycle sustaining chronicity and perhaps even achieve a cure for RA.

The immunosuppressive properties of MSCs are being exploited for the treatment of RA. It will be important to identify the RA patient subset most likely to respond to MSC therapy. Considering the presumed mechanism of action of MSCs to reset the immune system, an early intervention could be desirable. If patients receiving MSC-based therapy are already on conventional therapy such as DMARDs or biologics, then it will be essential to determine how these medications will alter MSC function. Experiments *in vitro* showed that the addition of TNFα, a key mediator in RA and one of the main targets of biological agents [2], reversed the suppressive effect of MSCs on T-cell proliferation [53,72]. MSC-based therapy in addition to anti-TNFα therapy could, therefore, have a synergistic effect in RA.

Systemically administered MSCs would represent a source of multipotent stem cells that could be available for the repair of damaged tissues while exerting their immunomodulation/suppression. The conflicting results in studies using MSCs emphasise the need for standardised and robust bioprocessing to obtain consistent and reliable MSC products. The development of *in vitro* assays of immunomodulatory function predictive of *in vivo* clinical outcomes will allow standardisation of MSC therapy and direct comparison between clinical studies. Other challenges relate to the biodistribution of the MSCs and their long-term fate in the body, which remain to be fully determined. Genetic engineering of MSCs for targeted migration to arthritic joints could be envisaged, for example, by MSCs expressing antibodies on their cell membrane that recognise epitopes specific to the damaged articular cartilage [73]. Ultimately, clinical studies will position MSC-based therapeutics in the treatment algorithm of RA, but this will also comply with individual patient characteristics, resulting in a personalised approach (optimal treatment at the right time in well-defined, stratified patients).

The success of the biologic agents targeting specific cytokines or cell types in the control of the inflammatory component of RA has made the biomedical community realise that other aspects of joint biology deserve more attention, such as the mechanisms driving tissue remodelling and repair. Established damage requires repair approaches and regenerative medicine offers potential for a lifelong solution. In orthopaedics, cell-based tissue repair has entered daily clinical practice, and there is anticipation that the development of injectable regenerative biologics will soon introduce this practice into rheumatology. Regenerative treatments will find applications for post-traumatic damaged joints, osteoarthritic and (post)-inflammatory joints and will include the repair of damaged joint surfaces or joint structures such as ligaments and menisci, or the implantation of off-the-shelf skeletal bio-structures, such as viable ligaments, menisci and other joint tissues.

In conclusion, MSC-based therapies via administration of exogenous MSCs or targeting of the endogenous MSCs in the joint are strategies that are being pursued to trigger/enhance repair of the damaged joint tissues, with the ultimate aim to restore joint homeostasis. With their wide range of functions, including immunomodulatory and anti-inflammatory properties, MSCs offer ample opportunities for the development of novel treatments for RA. This is an exciting journey in rheumatology and we are just at the beginning of it.

### Note

This article is part of a thematic series on *Biology and clinical applications of stem cells for autoimmune and musculoskeletal disorders*, edited by Christian Jorgensen and Anthony Hollander. Other articles in this series can be found at http://www.biomedcentral.com/series/MSC

## Abbreviations

DMARD: Disease-modifying antirheumatic drug
FLS: Fibroblast-like synoviocyte
GFP: Green-fluorescent protein
IFN: Interferon
IL: Interleukin
MSC: Mesenchymal stem cell
RA: Rheumatoid arthritis
TNF: Tumour necrosis factor
